# Early diagnosis of *Trichinella spiralis* and *Trichinella nativa*: Expression of the serine protease gene at the invasive intestinal and muscular larva stages

**DOI:** 10.14202/vetworld.2024.2124-2135

**Published:** 2024-09-20

**Authors:** Orken S. Akibekov, Aissarat M. Gajimuradova, Alfiya S. Syzdykova, Aibek Kh. Zhumalin, Fariza S. Zhagipar, Fabio Tosini, Zhannara Zh. Akanova, Nurtai N. Gubaidullin, Nasipkhan A. Askarova

**Affiliations:** 1 Department of Microbiology and Biotechnology, Faculty of Veterinary and Livestock Technology, Saken Seifullin Kazakh Agrotechnical Research University, 62 Zhenis Avenue, Astana 010000, Kazakhstan; 2 European Union Reference Laboratory for Parasites, lstituto Superiore di Sanità, 299, Viale Regina Elena, Roma 00161, Italy

**Keywords:** enzyme-linked immunosorbent assay, gene expression, invasion, transmembrane serine protease, *Trichinella nativa*, *Trichinella spiralis*, trichinellosis

## Abstract

**Background and Aim:**

Diagnosis of trichinellosis at the intestinal stage during larval development is the primary challenge in the early detection and treatment of trichinellosis. The use of serine protease as a diagnostic marker for serological tests has been the subject of various studies, but data on *Trichinella nativa* serine protease in the intestinal phase are still insufficient for a proper diagnosis. This study aimed to establish the duration of the intestinal phase for early diagnosis and to determine the level of expression of the serine protease gene in *T. nativa* and *Trichinella spiralis* larvae.

**Materials and Methods:**

We used European isolates from *T. spiralis* pigs and *T. nativa* larvae isolated from spontaneously infected wild carnivorous animals (wolf, Karaganda region) in Central Kazakhstan. Isolation of larvae from the meat of infected animals was carried out using the compressor method. For two species of *Trichinella*, 36 mice (in each group 18 mice) were infected with 250 larvae and euthanized by intramuscular injection of xylazine followed by an intravenous overdose of anestofol at 3, 5, 7, 14, 21, and 30 dpi (each day 3 infected mice) and one control group (3 mice). Sequencing and bioinformatics methods were used to determine the DNA and cDNA of the serine protease gene, and molecular methods (DNA extraction, reverse transcription polymerase chain reaction, and sequence) were used to measure the accumulation of serine protease transcripts in isolated larvae.

**Results:**

The results showed differences in the duration of intestinal phase between *T. spiralis* and *T. nativa*. The intestinal larvae of *T. nativa* were observed from 7 to 30 dpi, and the intensity of invasion increased up to 30 dpi (p < 0.001), while in the case of *T. spiralis*, the increase in larval growth in the intestinal phase decreased to 21 dpi, and only an increase of 1.6 ± 0.88 (p < 0.01) was detected at 30 dpi. *T. nativa* muscle larvae were detected at 21 dpi, compared with *T. spiralis* at 14 dpi. This characteristic was also reflected in the levels of serine protease transcripts in the samples. Accumulation was observed in both cases higher in the muscular stage of development, whereas the duration of the intestinal stage of *T. nativa* made it possible to detect serine protease at 30 dpi.

**Conclusion:**

The intestinal stage of *T. nativa* lasts for 30 days, indicating that the use of *T. nativa* serine protease is useful for the identification of intestinal infection. Furthermore, this protein can be used to identify *T. spiralis* and *T. nativa* in laboratory samples. Serine protease can be used as a marker for serological diagnosis. Within the framework of the research topic, it is important to conduct further studies on the species specificity of the obtained recombinant protein. It is necessary to focus on identifying highly specific *Trichinella* proteins for early disease detection.

## Introduction

Nematodes of the genus *Trichinella* are important food parasites that cause serious diseases worldwide. The main route of transmission from animals to humans is through consumption of infected meat [1]. The diagnosis of trichinellosis is also quite difficult since its signs and symptoms are non-specific [2]. Until now, the final diagnosis of human trichinellosis can only be made by detecting larvae in muscle biopsy or using highly specific immunodiagnostic tests, commonly indirect enzyme-linked immunosorbent assay (ELISA) tests. Excretory secretory antigens are the most commonly used diagnostic antigens of trichinellosis, but their main disadvantages are false-negative results at an early stage of infection, cross-infection, and the inability to recognize antibodies during the intestinal phase [3].

It is necessary to develop effective species-specific diagnostic medications to make an accurate diagnosis, determine the type of parasite, prescribe adequate therapy, and develop vaccines. In recent years, the creators of multi-epitope antigen-based vaccines for the control of schistosomiasis and malaria have achieved success. To date, scientists have indicated that it is necessary to include several antigens from various stages of the parasite’s life cycle in such vaccines [1]. Therefore, the identification of protein epitopes could facilitate diagnostic tests and the development of subunit vaccines.

For instance, in the life cycle of *Trichinella spiralis*, proteins excreted and secreted by muscle larvae (ML) and intestinal larvae (IL) are first exposed to the cells of the intestinal epithelium of the host, and they can play an important role in invading epithelial cells and causing an early immune response. Liu *et al*. [4] cultured ILs together with a monolayer of host epithelial cells and found that *Trichinella* larvae, penetrating the monolayer, produced various serine proteases. Serine proteases perform various biological functions during parasite invasion, migration, and proteolysis in host tissues and may be important antigenic molecular targets for *Trichinella* preparations [5].

Using proteomics and immunoproteomics methods, some serine proteases have been detected among the excretory-secretory (ES-Ag) or surface proteins of *T. spiralis* worms. Several types of serine proteases are involved in host epithelial cell invasion by infectious *T. spiralis* larvae. However, vaccination of mice with individual recombinant serine proteases only provided partial immunity against infection by *T. spiralis* larvae [6].

Yue *et al*. [7] identified *T. spiralis* serine proteinase (TsSerp; GenBank: AY028974.1) as one of the main ES proteins of *T. spiralis* ML by immunoproteomics; it was mainly localized in ML 30 days after infection (dpi).

Despite a sufficient number of publications on the serine protease of *T. spiralis*, limited information is available on the serine protease of *Trichinella nativa*. According to the NCBI database, the gene sequence has been sequenced only as part of the complete genome of the first chromosome (JYDW01000005), and its characteristics have not been described. The stages of dependent expression of the serine protease gene in *T. nativa* and *T. spiralis* have not been sufficiently studied for use as a diagnostic component for early trichinellosis diagnosis.

Given the insufficient knowledge of serine proteases in *T*. *nativa* species and the lack of information on the possibility of its use in the diagnosis of early stages of the disease in animals and humans, as well as the need to search for alternative proteins for the development of diagnostic drugs with proven stadium specificity, the purpose of this research was to study the characteristics of the *T. nativa* invasion process of *Balb/c* laboratory mice in a comparative aspect with *T. spiralis* at 3, 5, 7, 14, 21, and 30 dpi and determine the expression level of serine protease in *Trichinella* larvae at different developmental stages.

## Materials and Methods

### Ethical approval

All activities involving animals were carried out in compliance with high standards for biosafety and animal welfare. All protocols were implemented in accordance with the Ethical Guidelines for the Use of Animals in Research by the National Committee for Research Ethics in Science and Technology [8].

The Animal Ethics Committee of the Faculty of Veterinary Medicine and Animal Husbandry Technology of the NCJSC “S. Seifullin Kazakh Agrotechnical Research University” (KATRU), Astana, Kazakhstan (protocol no.1 from 23^rd^ of February 2023), approved the care and use of laboratory animals.

### Study period and location

This study was conducted from April-2022 to February-2023 on Research Platform of Agricultural Biotechnology and the Joint Kazakh-Chinese Laboratory for Biological Safety “Saken Seifullin Kazakh Agrotechnical Research University”.

### Source of *Trichinella larvae*

We used *T. nativa* larvae obtained from spontaneously infected wild carnivores. *T. spiralis* larvae were provided to us by a specialist in the Department of Diagnostics, Genetics and Characterization of the Pathogen of the Reference Center for Risk Assessment (BfR), Berlin (Germany), Dr. Anne Mayer-Scholl.

### Infection of animals

*T. nativa* and *T. spiralis* larvae were maintained through serial passaging of a Soviet chinchilla breed of rabbits at the Joint Kazakh-Chinese Laboratory for Biological Safety. The larvae were collected through artificial digestion using a standard protocol [9]. For each type of *Trichinella*, seven mice for 3^rd^, 5^th^, 7^th^, 14^th^, 21^st^, and 30^th^ days in 3–4 months age were selected for the experiment, and three mice were used as the control group. According to the principle of analogs, two groups of experimental animals of 18 mice per group were formed for *T. spiralis* and *T. nativa*. Each experiment was conducted 3 times to exclude the statistical error in the individuality of the immune system of each mouse model of infection. The causative agents of the two types of *Trichinella* invaded seven animals at a dose of 250 larvae per head. Animals were infected with oral *Trichinella* larvae using a disposable pipette.

### Experimental design

On the 3^rd^, 5^th^, 7^th^, 14^th^, 21^st^, and 30^th^ days, three mice from each group infected with *T. nativa* and *T. spiralis* were euthanized for pathoanatomic autopsy by intramuscular injection of xylazine (2 mg/kg bodyweight; Bioveta, Czech Republic) followed by an intravenous overdose of anestofol (15 mg/kg bodyweight; LLC “VIC”, Russia) on the 3^rd^, 5^th^, 7^th^, 14^th^, 21^st^, and 30^th^ dpi. The small intestines were removed during postmortem, opened longitudinally, and examined directly for presence of larvae following the recommendations of the WHO/OIE [10].

The muscles were examined for the parasite’s presence in accordance with Gamble *et al*. [11]. The small intestine was longitudinally dissected, washed three times with normal saline solution with ice, cut with sharp scissors into 2 cm long fragments, and cultured in normal saline solution at 37°C for 2.5 h. The larvae released from the small intestine into normal saline solutions were then collected using the Baermann method [12].

Diagnosis and isolation of the larvae of the causative agent of trichinellosis from animal muscle tissue samples were performed by compressor trichinoscopy and digestion in artificial gastric juice, in accordance with the methods of MUC 4.2.2747-10 “Methods of sanitary and parasitological examination of meat and meat products” [13]. The detected and isolated helminthological material was preserved in 70% ethanol solution.

### Antiserum testing using indirect ELISA

The determination of antiserum titers was performed according to the standard technique of the indirect ELISA [14] using polystyrene 96-well flat-bottomed tablets for ELISA (Corning, USA). The wells of the tablets were sensitized with *Trichinella* antigens at a concentration of 0.005 mg/mL and incubated at 4°C overnight. Antiserum samples (0.1 mL) were titrated in the wells of the tablet, starting with a dilution of 1:100 and incubated at 37°C for 60 min. The immune complex was detected using an antimouse immunoglobulin (Ig) G conjugate (Sigma, USA) and its substrate, tetramethylbenzidine (Sigma). To wash the solid phase from the non-binding components of the well reaction, the tablet was washed three times with bicarbonate solution (BCS) and three times with BCS-Tween). The ELISA results were assessed using a vertical light flow spectrophotometer (Bio San, China) at a wavelength of 492 nm. The extinction index was calculated for the antiserum titer, which is two or more times higher than the maximum value of the well in which the negative serum is titrated.

### Bioinformatics sequence analysis

Later, special programs were developed for testing using the Blast 11 version (https://blast.ncbi.nlm.nih.gov/Blast.cgi) and Primer Blast software (https://www.ncbi.nlm.nih.gov/tools/primer-blast/index.cgi?GROUP_TARGET=on) for three-phase *T. spiralis* and fully gene serine *T. nativa* translations. Empirical analysis was conducted using the Bepipred 3.0 program (https://biolib.com/DTU/BepiPred-3). Analysis based on scientific research identification of genetic origin was introduced into the CLC Sequence Viewer version 8.0 (Qiagen) software (https://clc-sequence-viewer.software.informer.com/8.0/).

### Molecular experiments

#### DNA extraction

Total genomic DNA was isolated using the traditional phenol-chloroform method, as described by Liu *et al*. [15]. Approximately 30 *Trichinella* larvae were placed in a 1.5 mL microcentrifuge tube and mixed with 500 μl of lysis buffer (50 mM Tris-HCl pH 8.0, 200 mM NaCl, 20 mM EDTA pH 8.0 and 1% SDS), 10 μL of proteinase K (20 mg/mL; Qiagen) were added, and the mixture was incubated at 55°C for 3 h. Proteinase K was inactivated at 100°C for 30 min. DNA was purified using phenolic and phenolic chloroform extraction and precipitated with ethanol. The DNA concentration was determined spectrophotometrically using a Nanodrop 2000 device (Thermo Fisher, USA). DNA was resuspended in 30 μL distilled water and frozen at –20°C for use in polymerase chain reaction (PCR).

#### PCR conditions

The reaction was carried out on a VeritiPro amplifier (Applied Biosystems, USA) using the following primers developed in this study:

For *T. spiralis* serine protease:

F: 5’—AATCGTCTTGTGGATG

CAAATGCAATAAC-3’;

R: 5’CTCTAGTGAAACCCC

AACCAGAAAGAAAAC-3’.

Product size 270 bp.

For *T. nativa* serine protease:

F: 5’- TTGCGGAACAGCCACAGTAG-3’;

R: 5’--CCTTAGGTCGCAATTCACCG-3’.

Product size 930 bp.

The composition of the reaction mixture (total volume 25 μL) included Taq 5X MasterMix (New England BioLabs, UK) - 5 μL, primer (10 mM) F-1 μL, primer R-1 μL, mQ water-15 μL, cDNA-3-3 μL (100 ng). PCR mode: primary denaturation at 95°C for 5 min. (1 cycle); denaturation at 95°C for 30 s., annealing of primers at 63°C (*T. nativa*) and 58°C (*T. spiralis*) for 30 s., elongation at 72°C for 60 s. (30 cycles); final elongation at 72°C for 5 min. (1 cycle).

### Sequencing

For sequencing, the resulting PCR product was isolated from an agarose gel using a Monarch DNA Gel Extraction Kit (New England Biolabs, England). Sequencing was performed using a SeqStudio genetic analyzer with the BigDye terminator 3.1 cyclic sequencing kit (ThermoFisher, USA). The reaction mixture consisted of a 3 mL BigDye terminator ready reaction mixture, DNA (100 ng/L) with a volume of 1 mL, primers (10 pmol/L) with a volume of 2 mL, and Milli-Q in a volume of 4 mL. The PCR program consisted of 25 cycles with initial denaturation at 96°C for 1 min, denaturation at 96°C for 10 s, hybridization at 50°C for 5 s, and elongation at 60°C for 4 min. The data were analyzed using the BLAST (https://blast.ncbi.nlm.nih.gov/Blast.cgi) and MEGA 11 (https://megasoftware.net) programs.

### Isolation of RNA

Total RNA was extracted from 5 IL and ML using TRIZOL reagent (Invitrogen, USA) [14] in accordance with the manufacturer’s instructions. The RNA concentration was measured using a NanoDrop 2000 (Thermo Scientific, USA).

### Reverse transcription

Total RNA was transcribed back into the first cDNA chain using a ProtoScript II First Strand cDNA Synthesis Kit (New England BioLabs) according to the standard protocol. To achieve this, a sample of isolated *Trichinella* RNA was mixed in a sterile microcentrifuge tube without RNase. The RNA samples were denatured for 5 min at 65°C. After mixing, they were left on ice. Next, 5× ProtoScript II Buffer 4 μL, 0.1 M DTT 2 μL, ProtoScript II RT (200 U/μL) 1 μL, RNase Inhibitor (40 U/μL) 0.2 μL, Nuclease-free H2O 2.8 μL were added. 20 μL of cDNA synthesis reaction was incubated at 42°C for 1 h. The enzyme was inactivated at 65°C for 20 min. The cDNA products were stored at –20°C.

### PCR conditions

The reaction was carried out on a VeritiPro amplifier (Applied Biosystems, USA) using the same primers. The product length of *T. nativa* cDNA was 691 bp without an intronic region included.

The primers for the cDNA of *T. nativa* were the same.

Reaction mixtures with a volume of 20 μL containing 2 μL DreamTaq Buffer (Thermo Fisher Scientific, USA), 2μL MgCI_2 (_2.5 mM), 1 μL dNTP (0.8 mM), 1 μL F, R-primer (20 pmol), 0.25 μL DreamTaq DNA polymerase, 1 μL cDNA (100 ng), and 11.75 μL H_2_O were prepared for the PCR. Conditions: The first cycle at 95°C for 3 min., the next 30 cycles of DNA denaturation at 95°C for 30 s., primer annealing at 63°C for *T. nativa*, 58°C for *T. spiralis* for 30 s. and DNA elongation at 72°C for 1 min., as well as the final cycle at 72°C for 7 min. Each PCR product was analyzed on a 1% agarose gel containing ethidium bromide [16].

### Statistical analysis

The average value and statistical error were calculated using Microsoft Excel 2010 (Microsoft Office, Washington, USA). Repeated measurement analysis of variance was used to determine differences between the numbers of larvae at different stages of invasion and between antibody levels. p < 0.05 was considered statistically significant.

## Results

### Invasion of laboratory mice by two *Trichinella species*

**Figure-1:**
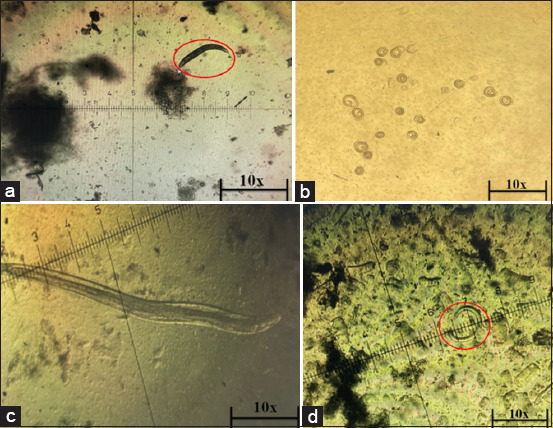
*Trichinella* larvae on the digestive tract and muscles in *Balb/c* mice: (a) *T. spiralis* larvae at 7 dpi, found in the intestines of mice, (b) *T. spiralis* larvae at 21 dpi, found in the muscles of mice, (c) *T. nativa* larvae at 7 dpi, found in the intestines of mice, and (d) *T. nativa* larvae at 21 dpi, found in the muscles of mice. *T. spiralis*=*Trichinella spiralis*, *T. native*=*Trichinella* *native*.

Infection with two types of *Trichinella* in laboratory mice revealed that these species have a species-specific invasive pathway in the body of mice. Thus, according to the results of the research, *T. nativa* has a less active and longer intestinal phase of infection, whereas the muscular stages of the larvae were detected 7 days later than in *T. spiralis*. Airas *et al*. [17] noted that after mating and reproduction, the first newborn larvae appear around the 5^th^ day after infestation. Table-1 presents quantitative data on larval detection in the intestines and muscles of laboratory mice. Figure-1 shows the larvae of two species of *Trichinella* isolated from IL and ML after 7 and 21 dpi.

**Table-1:** The numbers of *T. spiralis* larvae found in the intestines and muscles of mice.

Day after invasion	Number of days after invasion (dpi)					

	3	5	7	14	21	30
*T. spiralis*						
Intestine	-	-	9.3 ± 0.88	27 ± 1.73***	14 ± 2.08**	1.6 ± 0.88**
Muscles	-	-	-	61 ± 5.3	82 ± 4.73*	110 ± 4.3**
*T. native*						
Intestine	-	-	12 ± 1.53	15.7 ± 1.76^ns^	43.7 ± 3.28**	91 ± 3.61***
Muscles	-	-	-	-	17.7 ± 1.2	53.7 ± 1.45***
p-value IL			0.2	0.01	0.001	0.001
p-value ML					0.0002	0.0002

* p-values of IL and ML is determined between the species of *Trichinella*. *T. spiralis*=*Trichinella spiralis*, *T. native*=*Trichinella native*, ML=Muscle larvae, IL=Intestinal larvae

Infection by *T. nativa* IL was less intense than that by *T. spiralis* at 14 dpi. Although the number of *T. spiralis* ILs decreased by 21 dpi, the reproduction of *T. nativa* ILs increased by 2.9 times, although larvae of both species were found in muscle tissue. The number of *T. spiralis* ML was 3.5 times higher at 21 dpi than that of *T. nativa*. At 30 dpi, *T. nativa* infestation showed a 2-fold increase in the number of ILs compared to 21 dpi. At 30 dpi, 16 times fewer larvae were detected in *T. spiralis* than at 14 dpi, at which the maximum number of larvae was observed. This pattern was observed in all three repetitions of the experiment with a high level of confidence. In our opinion, this characteristic is species-specific. This could also be explained by the different stages of larval development during infection. However, larvae of both species were isolated from rabbit meat at 45 dpi, and infection was carried out in parallel on the same day. According to the results in pigs and rats, these two hosts are selective hosts of *T. nativa*, whereas in these hosts, *T. spiralis* causes a high intensity of infection (larvae per gram, lpg), and *T. nativa* is able to cause only minor infections [18]. The quantitative data obtained were confirmed for *T. spiralis*. According to Andreanov [19], the productivity of 100 *T. spiralis* larvae injected through the mouth showed survival in the intestine up to 18–24 days. Whereas Pereverzeva *et al*. [20] in a similar experiment did not observe intestinal sexually mature *Trichinella* in mice from day 21, and in the control group of mice, the survival rate of *Trichinella* IL was: after 4 days – 54%, 7 days – 44%, 15 days – 21%–30%, 21 days – 16.5%, and 28–30 days – 4%–5%, which is consistent with the results we have obtained. In the case of *T. nativa*, the literature data are very incomplete, and it was not possible to draw a reliable confirmation or refuting parallel between the scientific data. Perhaps, the lack of knowledge about *T. nativa* can be explained by the fact that this species is widespread among wild animals, unlike the “domestic” species of *T. spiralis*, which is actively studied due to its frequent infection of humans.

To determine the sensitivity of the serologic test at different levels of contamination, the serum of infected mice was tested using an indirect ELISA variant. Table-2 shows the results of the diagnosis of the two types of *Trichinella* at different stages of infection.

**Table-2:** Antibody titers of rabbits infected with *Trichinella* larvae against the ES-Ag of *T. nativa* and *T. spiralis* in indirect enzyme-linked immunosorbent assay.

Inventory/number of mice	Days after animal invasion						Mean antibody titers

	3 days	5 days	7 days	14 days	21 days	30 days	

	Antibody titers against ES-Ag *T. spiralis*						
1	PO	PO	PO	1:800	1: 1600	1:3200	1:1600 (+18.9; −15.9)
2	PO	PO	1:100	1:800	1:3200	1:6400	1:3200 (+20.6; −17.1)
3	PO	PO	PO	1:400	1:1600	1:3200	1:980 (+12.5; −11.1)
	Antibody titers of ES-Ag *T. nativa*						
1	PO	PO	PO	1:400	1:3200	1:6400	1:1970 (+20.6; −17.1)
2	PO	PO	PO	1:400	1:1600	1:3200	1:1210 (+17.3; −14.7)
3	PO	PO	PO	1:800	1:3200	1:6400	1:2430 (+17.3; −14.7)
	Control group						
7	PO	PO	PO	PO	PO	PO	-
8	PO	PO	PO	PO	PO	PO	-
9	PO	PO	PO	PO	PO	PO	-

*T. spiralis*=*Trichinella spiralis*, *T. nativa*=*Trichinella nativa*, ES=Excretory-secretory

The tested method showed low effectiveness in detecting trichinellosis IL in the intestinal phase of infection when larvae were already detected in the intestine. The effectiveness of the method was noted at 14 dpi at a titer of 1:400–1:800 in some mice from the group. In the case of *T. spiralis*, the larvae have already begun to be transported to skeletal muscles, making early medical therapy. For *T. nativa*, diagnosis at 14 dpi is not unambiguous at a titer of 1:400; it can be either a false positive or false negative. At 21 dpi, according to the titer, antibodies were detected in both cases of *Trichinella* infection. Despite the difference in the number of larvae, the titers were almost the same, which may also be due to individual susceptibility of the mice. The ELISA method is the main serologic method for detecting antibody titers to *Trichinella* in animals [21]. Therefore, early detection by serology and the development of specific antigens will allow the detection of trichinellosis and prevention of the spread of the disease and human infection. In recent studies, various *Trichinella* antigens have been used to increase the effectiveness of ELISA. For instance, the use of serine as an antigen for the detection of wild species such as *T. britovi, T. nativa, Trichinella* T6, and *T. pseudospiralis* in pigs has demonstrated significantly lower sensitivity for antibody detection, suggesting that it does not consistently detect exposure to forest *Trichinella* species [22].

### Molecular genetic analysis

**Figure-2:**
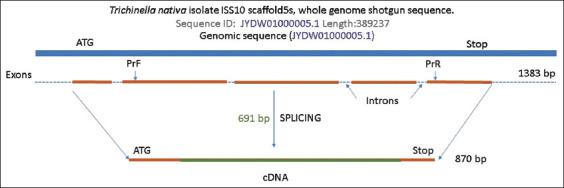
Diagram of the *Trichinella nativa* serine protease gene (based on the complete genome of the first chromosome).

After identifying the characteristics of the invasion of the two types of *Trichinella* using the example of laboratory mice, we identified the bioinformatic analysis of the study of the possibility of using serine protease in larvae for early diagnosis of the disease. For this purpose, we generated schemes for the *T. spiralis* and *T. nativa* genes based on available sequences in GenBank. Figure-2 shows a schematic of the *T. nativa* gene with the designation of translatable–exon and intron regions and their size.

**Figure-3:**
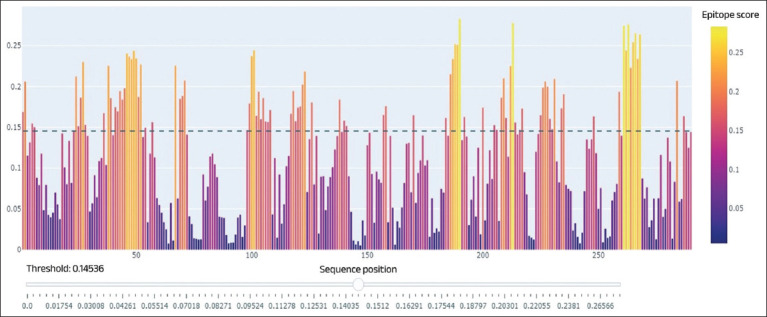
Epitope analysis of the Trichinella nativa serine protease gene site (Peaks located below 0.15 epitope score have a low probability of immune activity; peaks located above the specified value have a reliable probability of immune activity).

According to the gene scheme, most genes carry an information sequence. The gene includes five exons with short intronic regions between them, with a total length of 1.383 bp. The 870-bp coding region of the gene was used to design specific primers. Due to the impossibility of designing primers at the outermost regions of the first and last exons without the formation of hairpins and dimers, a central region with a length of 691 bp was chosen. For further use of this protein in serodiagnosis, it is important to identify the presence of epitope sites on the gene (Figure-3).

**Figure-4:**
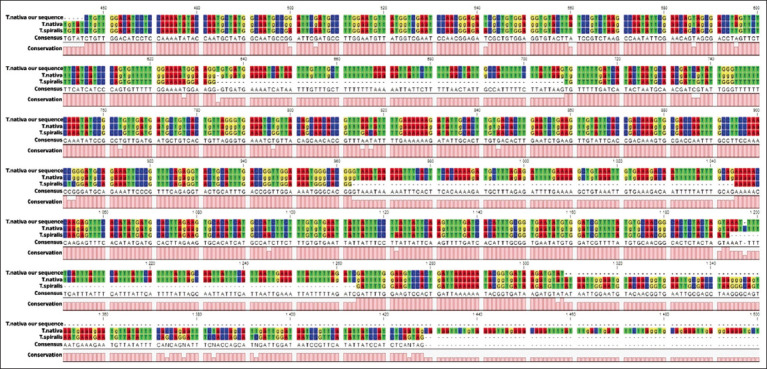
Alignment of the *Trichinella nativa* serine protease gene in the Blast program.

Epitope analysis revealed sufficient immunoactive sites, which allowed the selected sites to be used in further serologic analyses. The 930-bp gene has three large epitope sites and five sites with medium immunoreactivity. As it became clear during bioinformatics analysis, the nucleotide sequence of *T. nativa* serine protease is not presented in detail in genetic databases. During the research, primers were designed for sequencing the sequence of the serine protease gene, and further, its sequencing and alignment with similar gene sequences of other *Trichinella* species was carried out, where the sequence of the complete genome of the first chromosome of *T. nativa* was taken as a basis (Figure-4).

The nucleotide sequence of the *T. nativa* serine protease gene was sequenced and demonstrated to have two single-nucleotide deletions when aligned to the complete genome of chromosome 1 at positions 634 and 1997. Multi-nucleoid deletions and SNPs were observed in *T. spiralis*. The sequence of the *T. nativa* serine protease gene is registered in the NCBI database (number PP025901.1).

**Figure-5:**
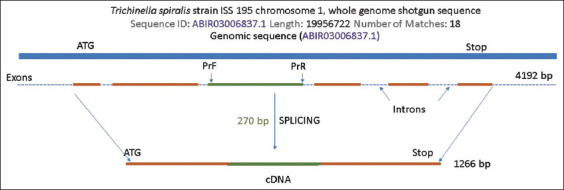
Diagram of the *Trichinella spiralis* serine protease gene (the section of the exon under study is highlighted in green).

**Figure-6:**
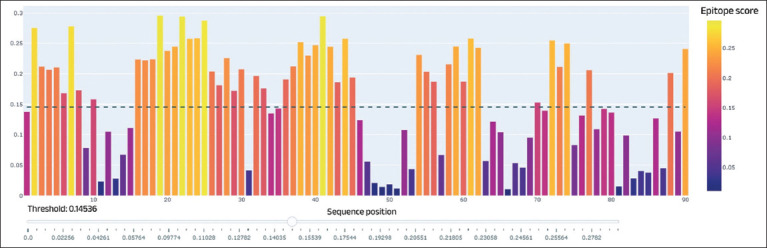
Epitope analysis of the *Trichinella spiralis* serine protease gene site (Peaks located below 0.15 epitope score have a low probability of immune activity; peaks located above the specified value have a reliable probability of immune activity).

**Figure-7:**
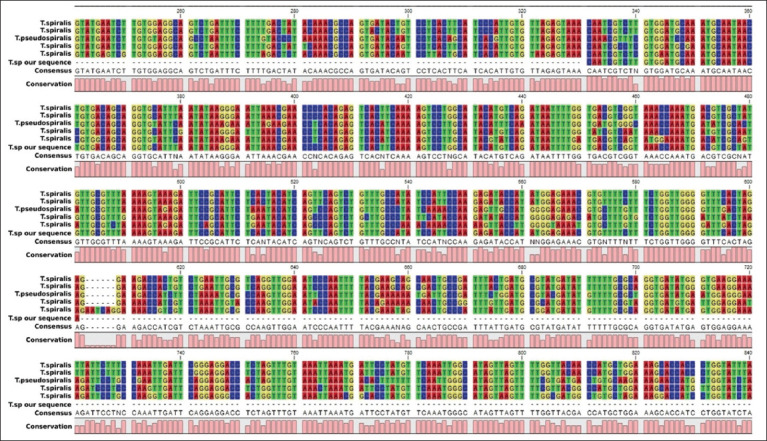
Alignment of the Trichinella spiralis serine protease gene in the Blast program.

A similar bioinformatics analysis was performed using the serine protease gene of *T. spiralis*. However, only the third exon of the gene was taken as the investigated region; since the gene has been sufficiently studied, it was important for us to identify the largest immunoactive region for further serodiagnosis. Figures-5-7 show a schematic of the gene, epitope analysis, and alignment of the sequenced regions.

The third exon is a large exon with high putative immunoreactivity. According to epitope analysis, this region contains up to five large epitope sites at 270 bp (Figure-6). Almost the entire site is immunoactive, thereby permitting its use in serological diagnostics. Using specific primers developed in this study, the sequence of the third exon was sequenced and aligned with the available reference sequences (Figure-7).

**Figure-8:**
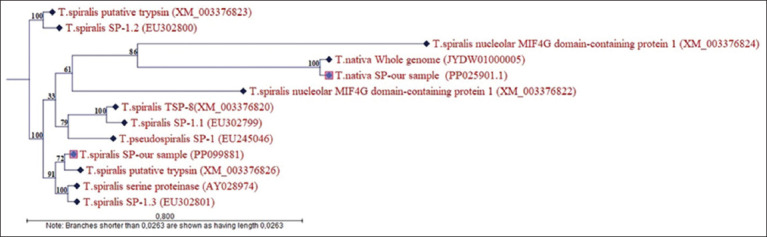
Phylogram of *Trichinella spiralis* and *Trichinella nativa* species based on the nucleotide sequences of the serine protease gene.

The resulting gene sequence was up to 100% similar to other sequences in the database. The nucleotide sequence of the registered section of the third exon is registered in the NCBI database under the number PP099881.1, based on which specific primers have been developed to determine the level of gene expression. Based on the obtained sequences, a phylogenetic tree was constructed using an algorithm to compare the relatedness of the selected serine protease gene regions of *T. spiralis* and *T. nativa* larvae with closely related *Trichinella* species available in the GenBank database. The branches indicate the identity (affinity) values of the studied serine protease gene sequences (Figure-8).

The maximum affinity (100%) of the *T. nativa* serine protease sequence was observed for the complete sequence of the first *T. nativa* chromosome. Among the sequences of the *T. spiralis* gene, affinity for the serine proteases of this species ranged from 72% to 100%.

**Figure-9:**
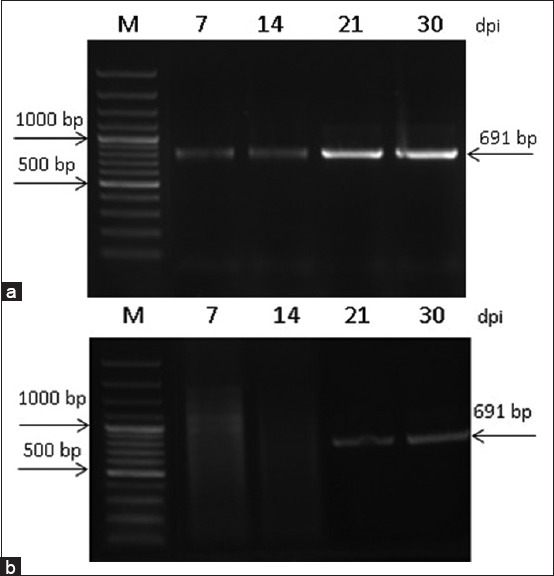
Numbers of transcripts of the serine protease gene and *Trichinella nativa* on 3, 5, 7, 14, 21, and 30 dpi in samples of (a) Intestinal larvae and (b) Muscle larvae.

**Figure-10:**
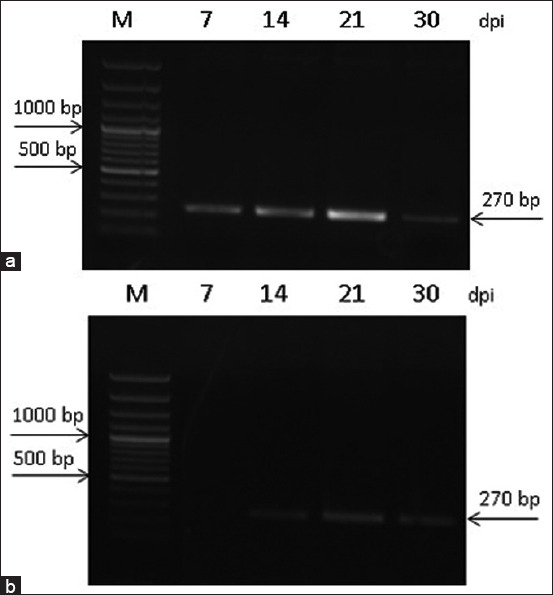
Number of transcripts of the *Trichinella spiralis* serine protease gene for 3, 5, 7, 14, 21, and 30 dpi in samples of (a) intestinal larvae and (b) muscle larvae.

Using the primers developed in this study, PCR of transcripts in *Trichinella* intestinal and muscular stages was performed (Figures-9 and 10).

The accumulation of transcripts was identified in this species only at the intestinal stage (7–30 dpi). Because of the duration of the intestinal phase, detection at this stage can be considered timely to prevent or reduce larval transport to the muscles. According to the dynamics of an increase in larval numbers to 30 dpi and transportation to muscles starting at 14 dpi, the transcripts of the gene was observed at 30 dpi. For ML, the detection of gene transcripts was also visualized at 21 and 30 dpi. However, the intensity of accumulation is significantly lower than in the IL. This result makes it possible to use serine protease in the diagnosis of the intestinal phase or its use as part of multicomponent vaccines and diagnostics. According to Wang *et al*. [23], who studied the serine protease of different *Trichinella* species, they detected the transcription and expression of the TspSP-1 gene.2 at all stages of *T. spiralis* development (adult worms, newborn larvae, precapsulated larvae and muscular larvae).

According to the results of gene expression analysis in *T. spiralis*, the picture was more active due to the intensity of infection (Figure-10).

From 7 dpi, the transcription of the serine protease gene in *T. spiralis* IL was almost at the same level. The accumulation of transcripts in *T. spiralis*, and *T*. *nativa*, was associated with the stage of larval transport into muscles. At 21 dpi, the maximum gene expression was observed. Despite a significant decrease in the number of larvae by 30 dpi, the expression level was relatively high. In these species, low levels of serine protease gene expression were detected in ML at 14 dpi. However, compared with *T. nativa* at 21 and 30 dpi, a less intense accumulation of transcripts was observed in ML. This may be related to the intensity of the encapsulation process of *T. nativa* ML. According to our earlier data [8], the muscle phase in *T. nativa* is characterized by a more intensive immune response, in particular, the production of neutrophils of more than 43% higher than in control, up to 70 dpi, which may explain the more active participation of serine protease in muscle encapsulation against the background of an active immune response. In the period from 14 to 21 dpi, the number of ML increased by 1.3 times. According to the intensity of the enzyme transcripts in *T. nativa* and *T. spiralis*, the main stages of larval transition from the intestinal to muscular stages can be distinguished. According to the literature, studies of *T. spiralis* proteases throughout the life of larvae show that excretory-secretory and crude extracts of muscle-stage larvae exhibit significant serine protease activity against structural proteins, whereas newborn larvae and adult worms predominantly destroy hematic proteins [24].

According to the data obtained, it is possible to identify the main stages of serine protease synthesis in the larvae of *Trichinella* of the intestinal and muscular stages of two species of *Trichinella*: T. *nativa* and *T. spiralis*, which allows us to expand data on the “wild” species of *Trichinella* of carnivorous animals that are ubiquitous in Kazakhstan [25].

## Discussion

Many proteases with different functions have been identified and characterized in parasitic nematodes. Since most of these classes of antigens are detected at the Ad and NBL stages [26], it is assumed that serine proteases play an important role in the development of *Trichinella* associated with the process of penetration into tissues [27], invasion [1–3], and migration [4, 5] of the parasite.

A previous study by Wu *et al*. [28] showed that serine proteases are involved in different events of the life cycle of parasites. The serine proteases of parasites are key factors in infection. Moreover, they are important enzymes involved in the ES production of *T. spiralis*. At least four types of serine proteases have been identified in *T. spiralis*, differing in their functions, localization, and molecular weight, and one of them is responsible for larval invasion at the intestinal stage (35.5 kDa) [5]. Many studies have shown that proteases have immunoprotective effects against *T. spiralis* infection in mice [29, 30]. Among these proteases, serine proteases play crucial roles in the invasion of *T. spiralis* into host cells and are involved in the evasion of the immune response [2, 31]. There is abundant evidence that serine proteases in the products of ES parasites perform many functions and are involved in feeding parasites and evading immunity [1–7, 32]. In addition, the participation of serine proteases in the survival of parasites has been demonstrated, and these proteases have been considered candidate antigens for vaccines against parasitic infections [3].

To identify the intestinal phase of development in the case of *T. nativa*, our study of the invasion characteristics of this parasite in laboratory mice showed that this species infects mice less intensively. Larvae were found in the intestine starting from 7 dpi, and their number increased up to 30 dpi from 12 to 91 larvae, whereas *T. spiralis* transformed into muscle tissues starting from 21 to 30 dpi, and the number in the intestine decreased significantly from 14 to 16 larvae. The identification of this pattern in the invasion of *T. nativa* larvae made it possible to identify the level of accumulation of larvae at each stage and to conduct a detailed study of the level of accumulation of transcripts of the serine protease gene.

Some serine proteases and their enzymatic activity (for example, Tdp SP1 and trypsin-like antigen 45 kDa) have been identified in *T. spiralis* ML and ES products [33, 34]. When ILs were co-cultured with host epithelial cells, the expression of serine protease in larvae at the intestinal stage of development was clearly increased compared to ML [35]. Another *T. spiralis* serine protease (Ts-Adsp) was tested based on the cDNA library of adult *T. spiralis*, and mice immunized with rTs-Adsp exhibited a 46.5% decrease in larval load compared with the control group [36]. Mice immunized with a DNA vaccine containing serine protease of newborn *T. spiralis* larvae showed a 77.93% decrease in the number of larvae after infection [37].

In this study, the expression level of serine protease was detected in *T. nativa* at 21 dpi, which, according to the refined invasion scheme in this species, makes it possible to detect the intestinal stage of larval development and conduct adequate targeted therapy. When infected with *T. spiralis*, the invasion process is more active than that in *T. nativa*; therefore, the expression of serine protease is observed at 14 dpi. The moment of expression detection is precisely associated with the transition of larvae from the intestinal to the muscular stage, which is consistent with early studies [3–5] and the characteristics of serine proteases as the main participants in the transition of larvae from the intestinal to the muscular stage. This aspect belongs to the two studied species; however, the peculiarities of invasion development do not allow identification of expression at 14 dpi. Identification of the expression level at the stage of ML in both species makes it possible to use this gene to specifically identify the parasite species without using multiplex PCR. It is worth noting that the level of transcript accumulation was significantly lower in *T. nativa* ML than in *T. spiralis* ML, which may be due to a more intense infection of the species.

The data obtained at the expression stage of serine protease in *Trichinella* will allow it to be used in the development of more accurate serological and PCR tests. Numerous attempts to improve the effectiveness of serological methods have led to the identification of several antigens of ML (antigen 53-kDa [38], glycoprotein 43-kDa [39], protein 45-kDa [40], TspSP-1 [41], Ts23-2 [42], serine proteinase inhibitor [43], protein P49 [44]); Ad (20 AD3 and 30 AD3 [45]) and NBL (a protein rich in glutamic acid [46]); recombinant cystatin-like protein (rCLP-cELISA) [47]; *T. spiralis* serine as a recombinant protein [22]; and recombinant protein rTs-CLP [26].

Previous studies by Xu *et al*. [48] and Gao *et al*. [49] have shown that the serine protease of adult *T. spiralis* larvae can participate in capsule formation and protect newborn larvae in the host circulatory system. These results suggest that serine proteases are associated with the invasion of the intestinal mucosa and can be potential targets for the early identification of parasite invasion and can be included in vaccines against *Trichinella* infection.

The duration of the intestinal stage of *T. nativa* allows the use of serine protease as an antigen to identify early infection, whereas in the case of *T. spiralis*, it is much more difficult to capture the intestinal stage before the introduction of ML due to the active development of infection. The use of serine protease in the early diagnosis of invasion will allow not only the detection of infection but also the use of serine protease as a component for the creation of multiprotein vaccines.

## Conclusion

The use of serine protease to detect the early intestinal invasion of *T. spiralis* and *T. nativa* is possible based on the transcripts identified starting from 7 dpi for *T. spiralis* and *T*. *nativa*. The invasion characteristics of the two types of *Trichinella* demonstrate that the intensity of larval accumulation in tissue samples is also important for detecting the presence of serine protease transcripts and determining the possibility of its use in early diagnosis. The results obtained will allow us to consider serine protease in the development of diagnostic tests for detecting *Trichinella* at the intestinal stage. It is also important to study the dose-dependent aspects of infection and the detection stages of protease transcripts.

## Data Availability

All data generated or analyzed during the study are included in this article.

## Authors’ Contributions

OSA: Designed and supervised the study and drafted the manuscript. AMG: Statistical analysis and drafted the manuscript. ASS, ZZA, and NAA: Designed and conducted the study. AKhZ: Bioinformatic analysis. FSZ: Conducted the study and drafted the manuscript. FT: Designed the study and bioinformatic analysis. NNG: Conducted the study and bioinformatic analysis. All authors have read, reviewed, and approved the final manuscript.

## References

[1] Yang Y, Vallee I, Lacour S.A, Boireau P, Cheng S.P, Liu M.Y (2015). Identification and characterization of immunodominant linear epitopes on the antigenic region of a serine protease in newborn *Trichinella* larvae. J. Helminthol.

[2] Wang L, Wang Z.Q, Hu D.D, Cui J (2013). Proteomic analysis of *Trichinella spiralis* muscle larval excretory-secretory proteins recognized by early infection sera. BioMed Res. Int.

[3] Xu D, Bai X, Xu J, Wang X, Dong Z, Shi W, Xu F, Li Y, Liu M, Liu X (2021). The immune protection induced by a serine protease from the *Trichinella spiralis* adult against *Trichinella spiralis* infection in pigs. PLoS Negl. Trop. Dis.

[4] Liu F, Song Y.Y, Zhang R, Liu R.D, Jiang P, Cui J, Wang Z.Q (2022). Cloning and expression of a new *Trichinella spiralis* serine protease and its role in invading host intestinal epithelium. Iran. J. Parasitol.

[5] Yang Y, Wen Y, Cai Y.N, Vallee I, Boireau P, Liu M.Y, Cheng S.P (2015). Serine proteases of parasitic helminths. Korean J. Parasitol.

[6] Ren H.N, Zhuo T.X, Bai S.J, Bai Y, Sun X.Y, Liu R.D, Long S.R, Cui J, Wang Z.Q (2021). Proteomic analysis of hydrolytic proteases in excretory/secretory proteins from *Trichinella spiralis* intestinal infective larvae using zymography combined with shotgun LC-MS/MS approach. Acta Trop.

[7] Yue X, Sun X.Y, Liu F, Hu C.X, Bai Y, Yang Q.D, Liu R.D, Zhang X, Cui J, Wang Z.Q (2020). Molecular characterization of a *Trichinella spiralis* serine proteinase. Vet. Res.

[8] Ethical Guidelines for the Use of Animals in Research (2019). National Committee for Research Ethics in Science and Technology (NENT).

[9] Akibekov O.S, Syzdykova A.S, Lider L.A, Zhumalin A.K, Baibolin Z.K, Zhagipar F.S, Akanova Z.Z, Ibzhanova A.A, Gajimuradova A.M (2022). Hematological, biochemical, and serological parameters of experimentally infected rabbits with *Trichinella nativa* and *Trichinella spiralis* for early identification of trichinellosis. Vet. World.

[10] Eckert J, Gemmel M.A, Meslin F, Paulowski Z, WHO/OIE (2001). Manual on Echinococcosis in Humans and Animals:A Public Health Problem of Global Concern.

[11] Gamble H.R, Alban L, Hill D, Pyburn D, Scandrett B (2019). International Commission on Trichinellosis:Recommendations on pre-harvest control of *Trichinella* in food animals. Food Waterborne Parasitol.

[12] Tintori S.C, Sloat S.A, Rockman M.V (2022). Rapid isolation of wild nematodes by Baermann funnel. J. Vis Exp.

[13] Committee of Sanitary and Epidemiological Control of the Ministry of Healthcare of the Republic of Kazakhstan (2020). Monitoring the Incidence of Parasitic Infections among the Population of the Republic of Kazakhstan.

[14] Supcharoengoon U, Reamtong O, Dekumyoy P, Watthanakulpanich D, Limpanont Y, Zhiyue Lv, Chaimon S, Martviset P, Adisakwattana P (2022). Evaluation of indirect-ELISA using eluted antigens from *Trichinella spiralis* muscle larvae for diagnosis of swine trichinellosis. Acta Trop.

[15] Liu A.W, Villar-Briones A, Luscombe N.M, Plessy C (2022). Automated phenol-chloroform extraction of high molecular weight genomic DNA for use in long-read single-molecule sequencing. F1000Res.

[16] Hasith Priyashantha A.K, Umashankar S (2021). Separation of DNA fragments using agarose gel electrophoresis;protocol, results, principle and possible errors to avoid. Adv. Biosci. Biotechnol.

[17] Airas N, Nareaho A, Linden J, Valo E, Hautaniemi S, Jokelainen P, Sukura A (2013). Early *Trichinella spiralis* and *Trichinella*
*nativa* infections induce similar gene expression profiles in rat jejunal mucosa. Exp. Parasitol.

[18] Bilska-Zajac E, Thompson P, Rosenthal B, Rozycki M, Cencek T (2021). Infection, genetics, and evolution of *Trichinella*:Historical insights and applications to molecular epidemiology. Infect. Genet. Evol.

[19] Andreanov O.N (2009). Biological Properties of the *Trichinella spiralis* Isolate of the Common Fox, Passioned on Laboratory Animals. Materials of the Scientific Conference Reports Theory and Practice of Combating Parasitic Diseases.

[20] Pereverzeva E.V, Ozereckovskaya N.N, Veretennikova N.L (1974). On the developmental particularities of *T.spiralis* larvae isolated from the racoon (*Procyon lotor*) muscles-in albino mice. Wiad Parazytol.

[21] OIE/World Organization for Animal Health Trichinellosis (Infection with *Trichinella spp*.) (2023). Manual of Diagnostic Tests and Vaccines for Terrestrial Animals 2023.

[22] Lobanov V, Konecsni K, Purves R, Scandrett W (2022). Performance of indirect enzyme-linked immunosorbent assay using *Trichinella spiralis*-derived Serpin as antigen for the detection of exposure to *Trichinella spp*. in swine. Vet. Parasitol.

[23] Wang B, Wang Z.Q, Jin J, Ren H.J, Liu L.N, Cui J (2013). Cloning, expression and characterization of a *Trichinella spiralis* serine protease gene encoding a 35.5kDa protein. Exp. Parasitol.

[24] Ros-Moreno R.M, Vazquez-Lopez C, Gimenez-Pardo C, De Armas-Serra C, Rodriguez-Caabeiro F (2000). A study of proteases throughout the life cycle of *Trichinella spiralis*. Folia Parasitol. (Praha).

[25] Akibekov O.S, Syzdykova A.S, Lider L.A, Zhumalin A.K, Zhagipar F.S, Gajimuradova A.M, Borovikov S.N, Suranshiyev Z.A, Ashimov S.A (2023). Trichinellosis dissemination among wild carnivores in the Republic of Kazakhstan:A 10-year study. Vet. World.

[26] Yamamoto M, Sawaya R, Mohanam S, Rao V.H, Bruner J.M, Nicolson G.L, Ohshima K, Rao J.S (1994). Activities, localizations, and roles of serine proteases and their inhibitors in human brain tumor progression. J. Neurooncol.

[27] Tang B, Liu M, Wang L, Yu S, Shi H, Boireau P, Cozma V, Wu X, Liu X (2015). Characterisation of a high-frequency gene encoding a strongly antigenic cystatin-like protein from *Trichinella spiralis* at its early invasion stage. Parasit. Vectors.

[28] Wu X, Fu B.Q, Wang X.L, Yu L, Yu S.Y, Deng H.K, Liu X.Y, Boireau P, Wang F, Liu M.Y (2009). Identification of antigenic genes in *Trichinella spiralis* by immunoscreening of cDNA libraries. Vet. Parasitol.

[29] Year H, Andiva S, Perret C, Limonne D, Boireau P, Dupouy-Camet J (2003). Development and evaluation of a Western blot kit for diagnosis of human Trichinellosis. Clin. Diagn. Lab. Immunol.

[30] Gamble H.R, Pozio E, Bruschi F, Nockler K, Kapel C.M, Gajadhar A.A (2004). International commission on Trichinellosis:Recommendations on the use of serological tests for the detection of *Trichinella* infection in animals and man. Parasite.

[31] Jung D, Teifke J.P, Karger A, Michael K, Venz S, Wittmann W, Kindermann K, Nockler K, Mundt E (2007). Evaluation of baculovirus-derived recombinant 53-kDa protein of *Trichinella spiralis* for detection of *Trichinella*-specific antibodies in domestic pigs by ELISA. J. Parasitol. Res.

[32] Hewitson J.P, Grainger J.R, Maizels R.M (2010). Helminth immunoregulation:The role of parasite secreted proteins in modulating host immunity. Mol. Biochem. Parasitol.

[33] Thawornkuno C, Nogrado K, Adisakwattana P, Thiangtrongjit T, Reamtong O (2022). Identification and profiling of *Trichinella spiralis* circulating antigens and proteins in sera of mice with trichinellosis. PLoS One.

[34] Romaris F, North S.J, Gagliardo L.F, Butcher B.A, Ghosh K, Beiting D.P, Panico M, Arasu P, Dell A, Morris H.R, Appleton J.A (2002). A putative serine protease among the excretory-secretory glycoproteins of L1 *Trichinella spiralis*. Mol. Biochem. Parasitol.

[35] Robinson M.W, Connolly B (2005). Proteomic analysis of the excretory-secretory proteins of the *Trichinella spiralis* L1 larva, a nematode parasite of skeletal muscle. Proteomics.

[36] Ren H.J, Cui J, Yang W, Liu R.D, Wang Z.Q (2013). Identification of differentially expressed genes of *Trichinella spiralis* larvae after exposure to host intestine milieu. PLoS One.

[37] Feng S, Wu X.P, Wang X.L, Bai X, Shi H.N, Tang B, Liu X, Song Y, Boireau P, Wang F, Zhao Y, Liu M (2013). Vaccination of mice with an antigenic serine protease-like protein elicits a protective immune response against *Trichinella spiralis* infection. J. Parasitol.

[38] Zarlenga D.S, Gamble H.R (1995). Molecular cloning and expression of an immunodominant 53-kDa excretory-secretory antigen from *Trichinella spiralis* muscle larvae. Mol. Biochem. Parasitol.

[39] Vassilatis D.K, Despommier D, Misek D.E, Polvere R.I, Gold A.M, Van der Ploeg L.H (1992). Analysis of a 43-kDa glycoprotein from the intracellular parasitic nematode *Trichinella spiralis*. J. Biol. Chem.

[40] Robinson M.W, Greig R, Beattie K.A, Lamont D.J, Connolly B (2007). Comparative analysis of the excretory-secretory proteome of the muscle larva of *Trichinella pseudospiralis* and *Trichinella spiralis*. Int. J. Parasitol.

[41] Wang L, Cui J, Hu D.D, Liu R.D, Wang Z.Q (2014). Identification of early diagnostic antigens from major excretory-secretory proteins of *Trichinella spiralis* muscle larvae using immunoproteomics. Parasit. Vectors.

[42] Nagano I, Wu Z, Nakada T, Boonmars T, Takahashi Y (2003). Molecular cloning and characterization of a serine proteinase gene of *Trichinella spiralis*. J. Parasitol.

[43] Nagano I, Wu Z, Nakada T, Matsuo A, Takahashi Y (2001). Molecular cloning and characterization of a serine proteinase inhibitor from *Trichinella spiralis*. J. Parasitol.

[44] Nash B, Gregory W.F, White R.R, Protasio A.V, Gygi S.P, Selkirk M.E, Weekes M.P, Artavanis-Tsakonas K (2023). Large-scale proteomic analysis of *T. spiralis* muscle-stage ESPs identifies a novel upstream motif for *in silico* prediction of secreted products. Front. Parasitol.

[45] Liu P, Wu X.P, Bai X, Wang X.L, Yu L, Rosenthal B, Blaga R, Lacour S, Vallee I, Boireau P, Gherman C, Oltean M, Zhou X.N, Wang F, Zhao Y, Liu M.Y (2013). Screening of early antigen genes of adult-stage *Trichinella spiralis* using pig serum from different stages of early infection. Vet. Parasitol.

[46] Zhuo T.X, Wang Z, Song Y.Y, Yan S.W, Liu R.D, Zhang X, Wang Z.Q, Cui J (2021). Characterization of a novel glutamine synthetase from *Trichinella spiralis* and its participation in larval acid resistance, molting, and development. Front. Cell Dev. Biol.

[47] Liu Y, Xu N, Li Y, Tang B, Yang H, Gao W, Liu M, Liu X, Zhou Y (2021). Recombinant cystatin-like protein-based competition ELISA for *Trichinella spiralis* antibody test in multihost sera. PLoS Negl. Trop. Dis.

[48] Xu J, Bai X, Wang L.B, Shi H.N, Giessen J.W.B, Boireau P, Liu M.Y, Liu X.L (2017). Immune responses in mice vaccinated with a DNA vaccine expressing serine protease-like protein from the new-born larval stage of *Trichinella spiralis*. J. Parasitol.

[49] Gao H, Tang B, Bai X, Wang L, Wu X, Shi H, Wang X, Liu X, Liu M (2018). Characterization of an antigenic serine protease in the *Trichinella spiralis* adult. Exp. Parasitol.

